# Environmental Metrics for Community Health Improvement

**Published:** 2010-06-15

**Authors:** Benjamin Jakubowski, Howard Frumkin

**Affiliations:** National Center for Environmental Health and Agency for Toxic Substances and Disease Registry, Centers for Disease Control and Prevention, Atlanta, Georgia, and Oberlin College, Oberlin, Ohio; National Center for Environmental Health/Agency for Toxic Substances and Disease Registry, Centers for Disease Control and Prevention

## Abstract

Environmental factors greatly affect human health. Accordingly, environmental metrics are a key part of the community health information base. We review environmental metrics relevant to community health, including measurements of contaminants in environmental media, such as air, water, and food; measurements of contaminants in people (biomonitoring); measurements of features of the built environment that affect health; and measurements of "upstream" environmental conditions relevant to health. We offer a set of metrics (including unhealthy exposures, such as pollutants, and health-promoting assets, such as parks and green space) selected on the basis of relevance to health outcomes, magnitude of associated health outcomes, corroboration in the peer-reviewed literature, and data availability, especially at the community level, and we recommend ways to use these metrics most effectively.

## Introduction

Metrics (or *indicators*) are powerful tools for tracking community health determinants and outcomes. Optimal metrics are measurable, simple, sensitive, robust, credible, impartial, actionable, and reflective of community values (1-3). Metrics can help identify problems, define community priorities, drive policy development, compare different communities, assess health disparities, and monitor progress over time in reaching goals.

Environmental metrics are a key part of the community health information base. Environmental factors greatly affect human health, both directly and proximately (eg, the quality of air people breathe) and indirectly and "upstream" (eg, the sources of energy a community uses). Environmental metrics may measure both unhealthy exposures, such as pollutants, and "salutogenic" exposures, such as parks and greenspace.

Three efforts help inform thinking about environmental metrics for community health. First, many communities identified *quality of life indicators* (also known as livability indicators) beginning in the 1980s (4). These frequently reflect environmental factors relevant to health. Second, *sustainability indicators* have recently found wide use (4). Many sustainability indicators pertain to environmental factors with clear relevance to human health (5). Third, the Council of State and Territorial Epidemiologists (6), collaborating with the Centers for Disease Control and Prevention (CDC), has addressed *environmental public health indicators*, emphasizing drinking water, air quality, asthma, and climate change.

We draw on each of these efforts to discuss environmental health metrics at the community level. Our logic model is based on the standard toxicologic sequence: exposure (in the environment) leads to dose (in the body), which leads to health effect. Since "exposure" can be either dangerous or salutary and either proximate or upstream, we consider several "exposure" metrics. These metrics fall into 4 major categories: measurements of contaminants in environmental media, such as air, water, and food; measurements of contaminants in people (biomonitoring); measurements of features of the built environment that affect health; and measurements of "upstream" environmental conditions relevant to health (Table). We selected metrics on the basis of relevance to health outcomes, magnitude of associated health outcomes, data availability (especially at the local level), and corroboration in the peer-reviewed literature. Finally, we discuss ways to integrate environmental data with other data and to apply them to public health action.

## Measurements of Contaminants in Environmental Media

Contaminants can be measured and tracked in air, water, and food, and waste production and exposure can be tracked via both emissions and residential proximity to waste sites.

Air pollution is associated with considerable illness and death. The Clean Air Act defines 6 "criteria pollutants" — carbon monoxide, nitrogen dioxide, ozone, sulfur dioxide, lead, and particulate matter (PM_2.5_ and PM_10_) — each with well-characterized health effects. Analysis of these pollutants is an established metric (6). The US Environmental Protection Agency (EPA) designates an additional 187 substances as hazardous air pollutants (HAP), which also threaten health (7). Although criteria pollutant levels are measured for regulatory purposes at approximately 5,000 sites nationwide, HAP monitoring is more sparse. These monitoring data are available through EPA's Air Quality System Data Mart (www.epa.gov/ttn/airs/aqsdatamart/), but poor temporal and spatial coverage and unrepresentative site placement limit their use. Communities can partially overcome these limits by using air quality modeling.

Water quality may be monitored both at the source (including groundwater and surface water) and at the tap. Metrics are available for both. The Clean Water Act requires states to monitor surface waters and to list those failing to meet water quality standards as "impaired" (8). A useful surface water quality metric is therefore the percentage of waters classified as impaired. Under the Safe Drinking Water Act, EPA has set national health-based standards for 90 microbiological, chemical, and radiologic drinking water contaminants in public water systems (9). Given this large number, metrics may include summary measures, such as annual number of drinking water contaminant exceedances and concentrations of selected indicator contaminants. Data are available through the EPA Safe Drinking Water Information System, including violation information for each public water system (www.epa.gov/safewater/databases/sdwis/index.html). Alternatively, data may be obtained directly from municipal water departments, which publish annual reports of water quality. Private wells and small water systems, which supply roughly 1 in 7 Americans with water, are exempt from routine monitoring (10).

Food contamination is measured on a national scale by the Food and Drug Administration (FDA) and the US Department of Agriculture (USDA). The FDA's Total Diet Study tests a market basket of 300 foods 4 times per year for pesticide residues, nutrient elements, industrial chemicals, and other chemical contaminants (11). The USDA tests agricultural commodities for pesticide residues through the Pesticide Data Program (www.ams.usda.gov/AMSv1.0/pdp) and verifies that pesticide tolerance levels established by the EPA are not violated in animal products through the National Residue Program (www.fsis.usda.gov/PDF/2009_Blue_Book.pdf). Regional or local monitoring of food contamination is rare (6); a unique exception is the measurement of contaminants in fish and shellfish in the Great Lakes (12). Food contaminants are not routinely measured at the community level, and feasible metrics have not been identified. However, an estimated 76 million illnesses are associated with microbiological food contamination each year (13), 44% of Americans eat at a restaurant on an average day (14), and local health departments routinely inspect restaurants. Therefore, the annual number of critical violations documented during restaurant inspections is a useful community metric.

Toxic chemical releases are tabulated by EPA's Toxic Release Inventory (TRI). This reporting system collects data on environmental releases of 581 chemicals and 30 chemical categories by facilities in selected industries, and the data are available online in EPA's TRI.NET system (www.epa.gov/tri/tridotnet/). The sum of annual toxic releases is a simple metric, but it fails to account for the variable toxicity of released chemicals. Communities can address this issue by using toxicity weighting tools (15). TRI data limitations include the 2-year time lag between toxic release and data release; the omission of thousands of chemicals in commercial production; reporting exemptions based on size, primary business activity, and chemical manufacturing, processing, and use thresholds; inaccuracies in self-reported data; and the fact that emissions do not equate to human exposures.

Hazardous waste exposure has been associated with self-reported poor health (16), decreased psychological well-being (17), and other health effects. Potential metrics include the number of hazardous waste sites in a community and the percentage of households living within 1 mile of a hazardous waste site, a distance at which health effects have been reported (18). Although data for such metrics are readily available through state environment departments, a limitation is that proximity to a waste site does not equate to human exposures.

## Measurements of Contaminants in People (Biomonitoring)

Biomonitoring, or measuring levels of contaminants in human samples (eg, blood, urine), is a powerful tool to quantify human exposure to chemicals and to link national risk assessments to specific community threats (19). CDC conducts ongoing biomonitoring on national population samples. Its *Third National Report on Human Exposure to Environmental Chemicals* reported blood and urine levels of 148 environmental chemicals (20), and the *Fourth National Report* added 75 new chemicals (21). Although the *National Report* does not provide data at the community level, it does provide national exposure levels that can serve as benchmarks for local comparison.

Lead screening in children is the only routine subnational application of biomonitoring. In 2006, more than 3 million children younger than 72 months had their blood lead levels checked (22). Communities may conduct other biomonitoring, especially if certain contaminants are of local concern; for example, 3 Minnesota communities with suspected exposures are measuring levels of arsenic, mercury, and perfluorochemicals (PFCs) under a biomonitoring pilot program (23). Such efforts can be complex and costly, up to $2,000 per person, depending on the analytes selected. Additionally, epidemiologic and toxicologic knowledge gaps frustrate efforts to translate exposure levels into health recommendations. Finally, although biomonitoring can unequivocally establish the occurrence of exposure, it is rarely useful in identifying its source.

## Measurements of the Built Environment

The built environment — places designed, shaped, and maintained by human activity — encompasses nearly all of the places we live, work, play, and study. It ranges from the small scale of rooms and buildings, to the intermediate scale of neighborhoods, to the large scale of metropolitan areas, and includes homes, sidewalks, parks, transit systems, roads, and more. The role of the built environment in health has been increasingly recognized in recent years (24). However, community health metrics of the built environment remain underused.

Automobile use is associated with air pollution, injuries and fatalities, physical inactivity, noise pollution, and other direct health effects (25), and contributes substantially to greenhouse gas (GHG) emissions (26). Reducing automobile use by reducing travel demand and shifting to alternative modes of transportation (eg, walking, bicycling, transit) can promote public health. Metrics of automobile dependence include average commute time to work and per capita daily vehicle miles traveled (DVMT). Annual county-level commute time data are available through the US Census Bureau American Community Survey (ACS) (www.census.gov/acs/www/index.html). The Texas Transportation Institute reports DVMT data for 90 US cities in its annual *Urban Mobility Report* (27); communities not included in the report can measure DVMT by using a survey instrument developed by the Energy Information Administration (28).

Measures of alternative transportation complement automobile dependence metrics. Public transportation use reduces automobile crashes, improves air quality, and entails routine physical activity (associated with walking to and from transit). Transit use can be measured as the proportion of employed people using transit to get to work; these data are collected in the ACS. Transit access can be measured as the proportion of households within 0.25 miles of a local bus or rail link, corresponding to the observation that people are willing to walk up to this distance to transit stops (29).

Other land-use and transportation features — population density, land-use mix, and connectivity (the ease of getting from one place to another, a function of the distance and directness of a trip route) — are associated with walkability, which in turn yields many health benefits. Population density can be calculated across spatial scales by using census data. Although measures of connectivity abound, average block length is often chosen because of its simplicity. Similarly, although many metrics of land-use mix are available (30), quantitative measures such as the index developed by Frank and Pivo (31) are frequently used. Distance between common trip origins and destinations also gives rise to some metrics. One example is the proportion of households with half-mile access to a public elementary school. This metric is relevant in relation to children's travel to school; during the past 30 years, the rate of active commuting has dramatically declined (32).

Because pedestrian infrastructure, such as sidewalks and trails, is associated with walking (33), metrics of this infrastructure, such as the ratio of sidewalk length to road length, are also salient. Unfortunately, data on sidewalk coverage are scarce, and data extracted from aerial photos are frequently of poor quality.

Bicycling complements walking by allowing active travel over greater distances. Bicycling infrastructure promotes bicycling (34); benefits include reduced body weight and reduced air pollutant and GHG emissions. Bicycle infrastructure can be measured as the length of the bikeway network, including bicycle paths and lanes, relative to total street miles.

Travel behavior, although it is not itself an environmental feature, offers metrics relevant to people's use of the built environment. The ACS measures the proportion of employed people who walk and bicycle to work. For children, active commuting to school can be measured by using parental surveys.

Green space, parks, and community gardens are examples of land use that promote health. Green space supports community health by reducing stress, promoting physical activity, and improving perceived general health (35). Percentage of tree canopy cover in an area is a widely used measure of community green space that can be determined through analysis of satellite or aerial images (36). Park access, a correlate of physical activity, can be measured as the proportion of households within 0.25 miles of a public park (sometimes limited to parks of a certain area, such as one-half acre or larger). Some communities measure the park and protected open space acreage per 1,000 residents. Finally, community gardens merit measurement because they benefit both gardeners and the public; increased physical activity, fruit and vegetable consumption, and community empowerment are all reported benefits of community garden programs (37). The proportion of households within 0.25 miles of a community garden and acreage used for community garden plots are metrics of community garden accessibility and density.

The food environment refers to the availability of both healthful and unhealthful foods in neighborhoods. Features of the food environment have increasingly been associated with eating patterns and nutritional status (38). However, practicable metrics of the food environment are only recently being developed and validated (39,40). Access to healthful food is a community asset. Full-service supermarkets provide more healthful food choices than do neighborhood groceries and convenience stores (39), and their presence has been associated with reduced overweight and obesity (41). Similarly, farmers' markets improve fruit and vegetable availability and provide a venue for education about healthful eating. In a longitudinal study of an African American community in North Carolina, establishing a community farmers' market significantly increased the proportion of residents who met daily fruit and vegetable consumption recommendations (42). The density of supermarkets in a census tract and the proportion of households within 1 mile of a farmers' market are metrics of a healthful food environment (43). Data supporting these metrics are available from local health departments and state agriculture departments, but geographic analysis is required.

Alcohol outlets, convenience stores, and fast-food restaurants are a counterpoint to supermarkets and farmers' markets. Studies have reported associations between alcohol outlet density and the prevalence of gonorrhea (44) and violence (45). Although distribution of alcohol licenses by zip code is a simple metric that uses publicly available data, finer geographic resolution is achieved by measuring the ratio of liquor outlets to roadway miles at the census tract level. Convenience store density and accessibility have been associated with increased prevalence of overweight and obesity (46-48); the corresponding metric is census tract convenience store density. Although an association between fast-food accessibility and obesity has not been observed in the general population, children and adolescents may be at risk. Elevated densities of fast-food restaurants have been reported around schools in Chicago (49) and Los Angeles (50), and some Californian middle- and high-school students attending schools located within 0.5 miles of the nearest fast-food restaurant are more likely to be obese or overweight than their counterparts attending schools in environments with more healthful foods (51). On the basis of these findings, the number of schools located within 0.5 miles of a fast-food restaurant may be a useful metric.

## Moving Further Upstream

Some environmental practices and features affect health indirectly, over large spatial scales, and over long periods. Such factors are not typically considered as community health metrics but may be informative and may help define community health aspirations and plans.

Development and use of renewable energy resources can mitigate climate change, reduce air pollution, and eliminate diseases and injuries associated with fossil fuel extraction (52). The corresponding metric is the proportion of electricity derived from renewable sources, drawing on data available from local utilities. Annual per capita GHG emissions (53) is a related metric. One approach to this metric is calculation of the "carbon footprint," and many "carbon footprint calculators" are available (http://co2list.org/files/calculators.htm). Such calculations are complex; transportation, dietary habits, electricity production, natural gas consumption, and landfill waste decomposition must all be considered. Regardless, the potential health effect of climate change supports use of this metric.

Metrics of waste management are relevant both because waste can have an effect on public health, and waste generation indirectly reflects resource depletion. Two metrics suitable for use at the community level are the proportion of the waste stream diverted from landfill and annual per capita quantity of landfilled solid waste. Resource depletion goes well beyond waste generation, to include biodiversity loss, soil erosion, groundwater depletion, and other aspects of environmental degradation (54), but no feasible community-level measures of these long-term health determinants have been identified.

## Health Effects Associated With Environmental Exposures and Environmental Policies for Health

Although measures of general health outcomes are discussed elsewhere in this issue of *Preventing Chronic Disease (PCD)*, some diseases deserve mention here because of their close associations with environmental exposures (6). One category is diseases uniquely related to environmental exposures; examples include pesticide toxicity (from pesticides) and asbestosis and mesothelioma (from asbestos). The incidence of these diseases may be a useful metric in populations with known exposure risks. A second category is diseases with complex causes, including a substantial environmental component, such as asthma and hearing loss. The incidences of such conditions may be useful environmental health metrics, but they must be interpreted cautiously because other etiologic factors play important roles.

Similarly, although health policies and programs are addressed elsewhere in this issue of *PCD*, policies that reduce community exposures to environmental hazards deserve mention here (6). The prototypical environmental health policy is enforceable limits on smoking in public places, but policies ranging from zoning ordinances to open burning bans can promote health and may provide useful metrics.

## Integrating and Applying Environmental Data for Public Health

Environmental metrics provide valuable information, and when combined with other community health metrics can help identify problems, define priorities, inform policy development, compare different communities, assess health disparities, and monitor progress over time in reaching goals. Environmental metrics must be applied strategically to maximize their effect on public health. This approach requires appreciating differences among communities, using techniques (eg, geographic information systems [GIS]) to connect environmental data with communities, and applying metrics toward policy making.

Not every environmental metric of community health is applicable to every community. Demographic and geographic differences matter (55). For example, coastal water quality indicators are regionally specific; a northwest community may measure Chinook spawning in local waterways, whereas an Atlantic coastal community may measure harvestable shellfish beds. Involving communities in defining and using metrics can help ensure metric relevance and promote long-term program sustainability (56,57).

Metrics are data, and data must be integrated to yield information. An invaluable tool is GIS, which helps link health determinants and outcomes over appropriate spatial scales. GIS not only allow for data integration but also facilitates communication between the public and professionals by providing a common language, namely the language of place (58). GIS is also ideally suited to identify health disparities and environmental injustices in communities (59). By integrating environmental measurements with demographic information, including race, ethnicity, and socioeconomic status, inequities can be identified, and interventions can be directed to improve the health of disenfranchised populations. GIS also facilitates public education, a commonly cited goal of community indicator projects. However, realizing the educational value of community measurements requires supplementing visual information with plain language translations of technical metrics and synthesis of broader narratives that reconnect with community values. Because of GIS's emerging emphasis, GIS capacity is increasingly an essential part of community metrics.

Finally, metrics must be used to drive policy and achieve and reward sustained community health improvements. This approach requires engaging decision makers in indicator development (60) and tying policy initiatives to metrics. For example, the San Francisco Bay Area's transportation plan, *Transportation 2035: Change in Motion*, establishes targets for reduced emissions of carbon dioxide, PM_2.5_ and PM_10_, per capita vehicle miles traveled, and travel delay (61). Environmental metrics of community health can and should be tied to health and health equity targets to maximize their ability to improve community health and well-being.

## Figures and Tables

**Table.** Environmental Metrics for Community Health Improvement

Environmental Factor	Metric	Magnitude of Health Effect^a^	Ease of Use/Data Collection^a^	Ability to Detect Disparities^a^
**Measurements of contaminants in environmental media**				
**Air quality**	Criteria pollutant levels^b^	1	1	2
	Hazardous air pollutant levels	1	3	3
**Water quality**	Percentage of surface waters listed as "impaired"^b^	2	1	2
	Number of drinking water contaminant exceedances^b^	1	1	2
	Concentrations of drinking water contamination indicator contaminants^b^	1	1	2
**Food contamination**	Annual number of critical violations during routine restaurant inspections	2	1	2
**Toxic releases**	Environmental releases of Toxic Release Inventory chemicals by reporting facilities^b^	2	1	1
**Hazardous waste**	Percentage of households living within 1 mile of a hazardous waste site	3	1	1
**Measurements of contaminants in people**				
**Biomonitoring**	Prevalence of elevated blood lead levels in children^b^	1	1	1
**Measurements of the built environment**				
**Transportation and land use**	Percentage of employed persons riding public transit, walking, and bicycling to work	2, 1, 1	2, 2, 2	2
	Average commute time to work	2	1	2
	Per capita daily vehicle miles traveled^b^	2	3	2
	Population density	2	1	1
	Connectivity (ease of traveling between 2 points): average block length	2	2	1
	Land-use mix (diversity of land uses [eg, residential, commercial, recreational, educational] within a defined area)	2	3	1
	Percentage of households within 0.25 miles of a local bus or rail link^b^	2	2	1
	Ratio of sidewalk length to road length	2	2	1
	Length of bikeway network relative to total street miles	2	2	2
	Percentage of households within 0.5 miles of a public elementary school	2	2	1
	Active commuting rates in school children	1	2	2
**Green space, parks, and community gardens**	Percentage households within 0.25 miles of a public park one-half acre or larger	1	2	1
	Park and green space acreage per 1,000 residents	2	1	1
	Percentage of tree canopy cover in an area	2	2	1
	Percentage of households within 0.25 miles of a community garden	2	2	1
	Acreage used for community garden plots	3	2	2
**Food environment**	Percentage of households within 1 mile of a farmers' market	2	2	1
	Supermarket density	1	2	1
	Alcohol license density	2	2	3
	Ratio of liquor outlets to roadway miles	1	3	1
	Convenience store density	1	2	1
	Number of schools within 0.5 miles of a fast-food restaurant	2	2	1
**Measurements of upstream factors relevant to health**				
**Environmental conditions **	Percentage of electricity from renewable sources (eg, wind, solar, geothermal)	2	2	3
	Annual per capita greenhouse gas emissions	1	3	3
	Percentage of waste stream diverted from landfill	2	2	3
	Annual per capita landfilled solid waste	2	2	3

a Scores from 1 to 3 are semi-quantitative assessments based on the authors' assessments, reached by agreement of the 2 authors, with 1 being greatest.

b From the Council of State and Territorial Epidemiologists (6).
